# Rapid cyclic fluctuations in blood pressure during two surgeries associated with pheochromocytoma: a case report

**DOI:** 10.1186/s40981-022-00569-6

**Published:** 2022-10-05

**Authors:** Tomoyuki Watanabe, Hirotsugu Miyoshi, Ryuji Nakamura, Sachiko Otsuki, Noboru Saeki, Yasuo M. Tsutsumi

**Affiliations:** Department of Anesthesiology and Critical Care, Hiroshima University, 1-2-3 Kasumi, Minami-ku, 734-8551 Hiroshima, Japan

**Keywords:** Pheochromocytoma, Periodic blood pressure fluctuations, Catecholamine

## Abstract

**Background:**

We measured catecholamine levels during periodic blood pressure fluctuations in patients with pheochromocytoma.

**Case presentation:**

A 43-year-old man presented with periodic blood pressure fluctuations during surgery for a renal pelvic tumor. His blood levels of catecholamines (ng/mL) changed dramatically over a short time during blood pressure fluctuations: adrenaline 0.36 to 3.22, noradrenaline 0.47 to 1.98, and dopamine 0.02. After the diagnosis of pheochromocytoma, oral treatment with doxazosin 2 mg/day was administered, and left adrenalectomy was performed 4 months after the initial surgery. Periodic circulation fluctuations occurred after tracheal intubation at the time of anesthesia induction, but the degree of fluctuation was smaller than that of the first surgery.

**Conclusions:**

The data suggest that the periodic blood pressure fluctuations in pheochromocytoma patients are caused by changes in blood catecholamine levels. Our data suggests that alpha blockers may also be effective against the cyclic fluctuations that occur in patients with pheochromocytoma.

## Introduction

Pheochromocytoma is a disease that exhibits various symptoms, such as hypertension, hypermetabolism, and hyperglycemia, due to abnormal catecholamine production by the tumor. It is well known that pheochromocytoma causes transient abnormal hypertension due to physical pressure on the tumor; however, there are few reports to date of periodic blood pressure fluctuations [1]. The cause of these fluctuations remains unknown [2, 3]. Some reports have speculated that periodic changes in blood pressure occur either in the case of sympathetic reflex or central nervous system ischemic reflex. They have also shown that stabilizing circulating blood volume may be the solution to this anomaly that interrupts the circulating cycle [2]. It is still unclear whether the pathophysiology of periodic blood pressure fluctuations in patients with pheochromocytoma is associated with this sympathetic and parasympathetic reflex cycle or the central nervous system ischemic reflex [4, 5].

We report a case of pheochromocytoma with periodic abnormal circulatory fluctuations during two surgeries. We found that blood catecholamine levels fluctuated during the periodic blood pressure fluctuations. Written and informed consent has been obtained from the patient for the publication of this case report.

## Case description

Our patient was a 43-year-old man with a weight of 93 kg and height of 174 cm. He had been undergoing hemodialysis for 20 years because of chronic renal failure. He had been taking cinacalcet, calcium carbonate, montelukast, loratadine, and nalfurafine before the surgery. There were no preoperative symptoms of suspected pheochromocytoma and no history of hypertension. His blood pressure was 140/82 mmHg, heart rate 76 beats/min, body temperature 36.2 °C, and normal respiratory rhythm with an O_2_ saturation of 96% on room air. Blood tests demonstrated BUN of 60.2 mg/dL and creatinine of 13.3 mg/dL, but coagulability, liver function, and electrolytes were normal.

**Fig. 1:**
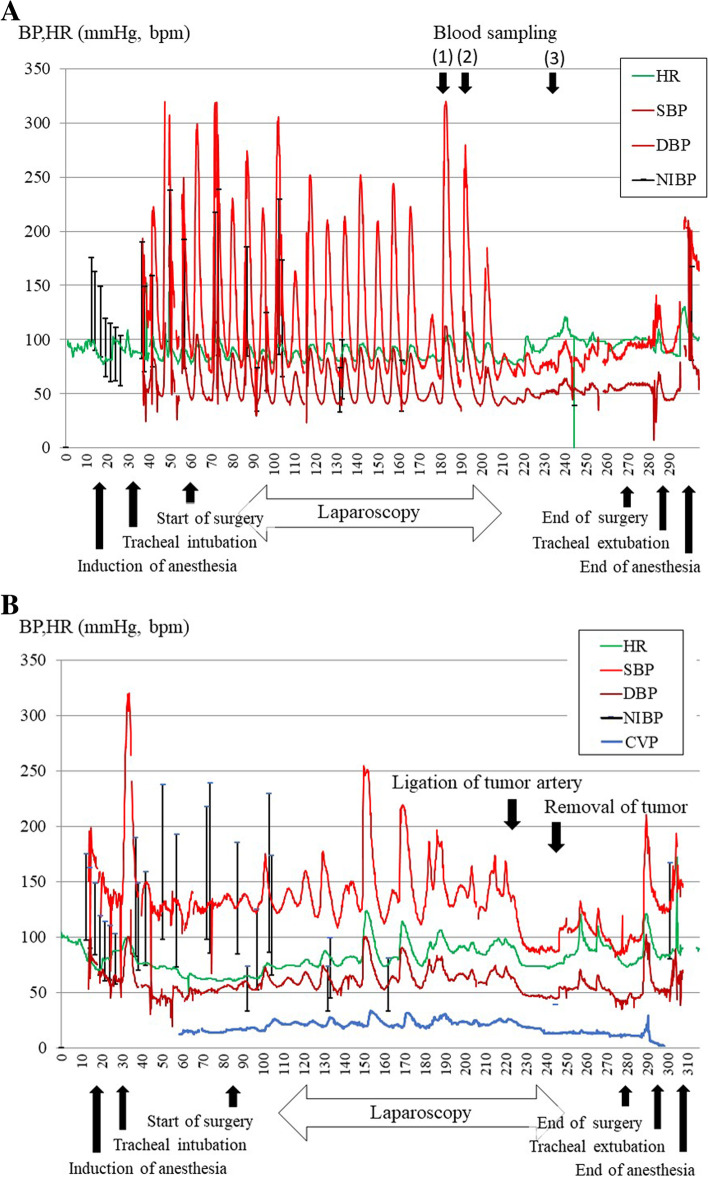
Anesthesia record of the first (**A**) and the second surgery (**B**). **A** Periodic blood pressure fluctuations occurred after tracheal intubation, lasted approximately for 150 min, and subsided at the end of laparoscopic surgery. Levels of adrenaline, noradrenaline, and dopamine were 3.22, 1.98, and 0.02 ng/ml at the point of [1], 6.32, 3.12, and 0.02 ng/ml [2] and 0.36, 0.47, and 0.02 ng/ml [3], respectively. **B** Periodic blood pressure fluctuations occurred after anesthesia induction, lasted approximately for 200 min, and disappeared after pheochromocytoma resection. The fluctuations were approximately 10 min apart, and the degree was smaller than that of the first surgery

**Fig. 2:**
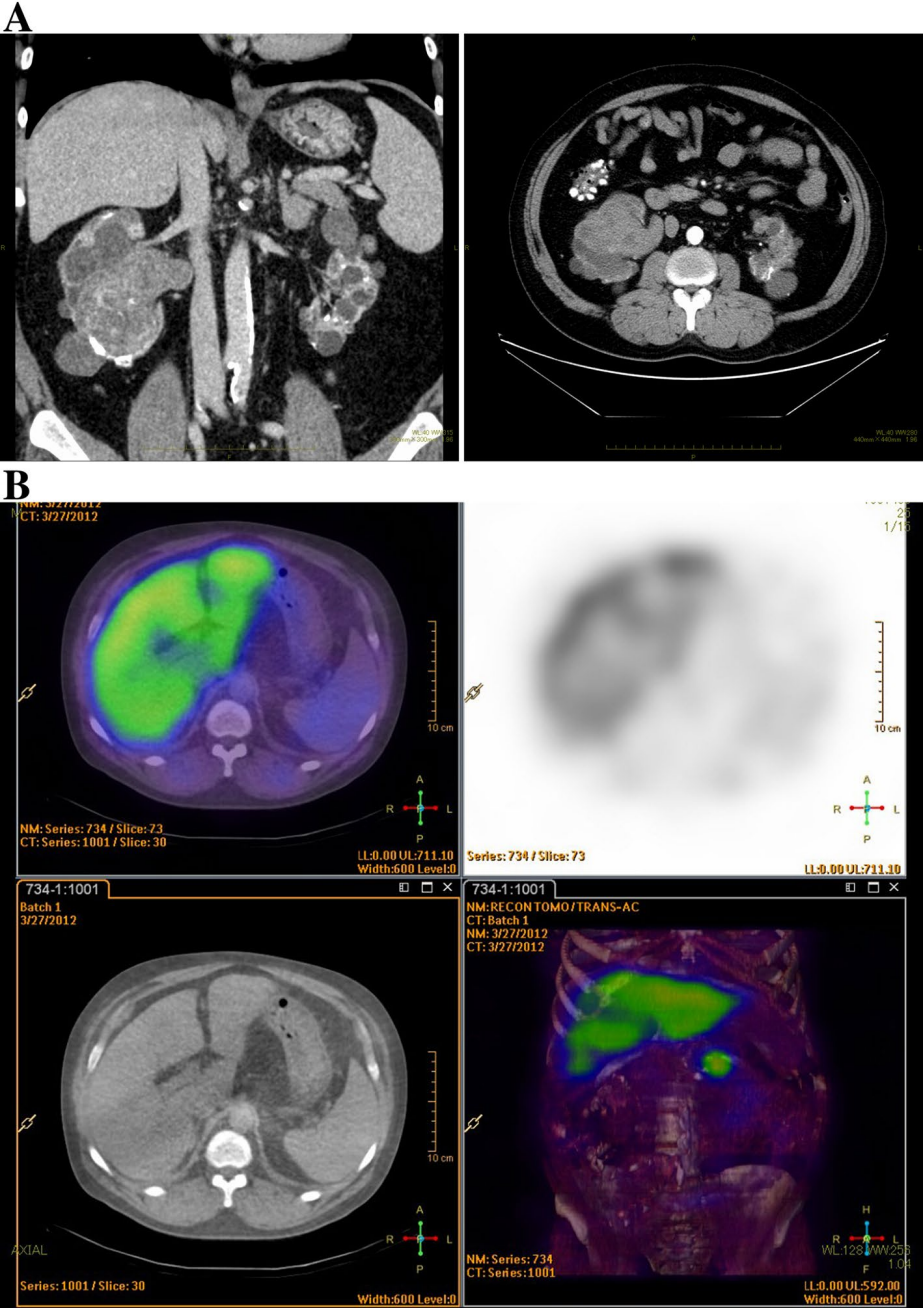
Abdominal computed tomography (**A**) and ^131^I-metaiodobenzylguanidine scintigraphy images (**B**). **A** Computed tomography showed multiple renal cysts in both kidneys, left kidney atrophy, and right renal pelvis tumor (64 × 55 mm). **B** Scintigraphy identified an increased uptake of ^131^I-metaiodobenzylguanidine corresponding to the adrenal tumor

A close examination of hematuria revealed a tumor in the right renal pelvis, and retroperitoneal total right nephroureterectomy was scheduled. The tumor in the right adrenal gland was followed up because there was no clear malignant finding on CT. On the day before surgery, 5100 mL of body fluid was removed by hemodialysis. Propofol and remifentanil were used for the induction and maintenance of anesthesia. After tracheal intubation, periodic systolic blood pressure fluctuations within the range of 70–260 mmHg, diastolic blood pressure fluctuations within the range of 40–90 mmHg, and heart rate of 80–110 beats/min were observed at intervals of approximately 8 min (Fig. 1A). Intermittent and continuous administration of nicardipine was not effective for suppressing the fluctuation, which lasted for approximately 3 h and disappeared spontaneously after completion of laparoscopic surgery. Intermittent and continuous administration of nicardipine was performed because of this circulatory fluctuation; however, it could not be suppressed. Periodic blood pressure fluctuations disappeared spontaneously at the end of laparoscopic surgery. Blood samples were collected during the cycle of circulatory fluctuations. The catecholamine levels of the blood samples were as follows: [1] first blood pressure fluctuation: adrenaline 3.22 ng/mL, noradrenaline 1.98 ng/mL, and dopamine 0.02 ng/mL; [2] second blood pressure fluctuation: adrenaline 6.32 ng/mL, noradrenaline 3.12 ng/mL, and dopamine 0.02 ng/mL; and [3] at the end of blood pressure fluctuation: adrenaline 0.36 ng/mL, noradrenaline 0.47 ng/mL, and dopamine 0.02 ng/mL. A postoperative examination revealed that the highest blood catecholamine levels were adrenaline, with 0.03 ng/mL (normal value: 0–0.10 ng/mL); noradrenaline, with 0.52 ng/mL (normal value: 0.10–0.50 ng/mL); and dopamine, with 0.03 ng/mL (normal value: 0–0.03 ng/ml). In addition, scintigraphy identified an increased uptake of ^131^iodine-metaiodobenzylguanidine corresponding to the left adrenal gland, suggesting pheochromocytoma (Fig. 2). After the diagnosis of pheochromocytoma, oral treatment with doxazosin 2 mg/day was administered, and left adrenalectomy was performed 4 months after the initial surgery.

Before surgery, his blood pressure was 170/92 mmHg, heart rate 80 beats/min, body temperature 36.5 °C, and normal respiratory rhythm with an O_2_ saturation of 96% on room air. Blood tests demonstrated Hb of 10.4 g/dL, BUN of 46.9 mg/dL, and creatinine of 11.6 mg/dL, but coagulability, liver function, and electrolytes were normal. Echocardiography showed left ventricular hypertrophy and mild left ventricular wall hypokinesis. On the day before surgery, 4300 mL of body fluid was removed by hemodialysis. Pheochromocytoma resection was performed under general anesthesia with propofol and remifentanil. As with the initial surgery, periodic circulation fluctuations occurred after tracheal intubation at the time of anesthesia induction, but the fluctuations were approximately 10 min apart, and the degree of fluctuation was smaller than that of the first surgery. Intermittent and continuous administration of nicardipine and landiolol was performed for circulatory fluctuation; however, once again, the fluctuations could not be suppressed. Dopamine was administered to maintain circulation after tumor removal. The periodic fluctuations disappeared after the ligation of the left adrenal artery and did not occur thereafter. The intraoperative course of the second surgery is shown in Fig. 1 (Fig. 1B).

## Discussion

Pheochromocytoma is a catecholamine-producing tumor, and abnormal hypertension, one of its symptoms, is generally divided into paroxysmal, persistent, and mixed types [6, 7]. There are few reports of cyclical fluctuations of blood pressure in patients with pheochromocytoma during awake condition [8] and under anesthesia [9], but no reports describe such fluctuations recurred during repeated surgeries as observed in our case. The pathophysiology of this blood pressure cyclical fluctuation is unknown; however, baroreceptors respond to a rapid rise in blood pressure during an acute attack, activating a negative feedback loop through both the sympathetic and parasympathetic nervous systems [8, 9]. A presumed result is a sharp rise and fall in blood pressure. We found that blood catecholamine levels fluctuated significantly according to blood pressure fluctuation, and it decreased when the blood fluctuation disappeared. Furthermore, the blood pressure fluctuation disappeared after removal of the tumor, suggesting that circulation fluctuation was caused by catecholamine released by the pheochromocytoma.

Wave-like patterns of blood pressure changes can be produced in laboratory animals under certain conditions and are called Mayer waves. This is likely due to reflex mechanisms involving either chemoreceptors or baroreceptors [4]. These physiological reflexes may be accentuated in pheochromocytoma. It is not known whether periodic abnormal blood pressure fluctuations which occur in patients with pheochromocytoma are caused by enhancement of physiological reflexes by tumor-released catecholamines or by cycles of tumor-released catecholamines themselves. However, the fact that periodic blood pressure fluctuations disappeared after tumor resection in our patient suggests that catecholamines were periodically released from the tumor.

The trigger for the periodic cyclic fluctuations was unknown. In general, the causes of catecholamine release associated with pheochromocytoma include tumor blood flow, physical compression, tumor tissue collapse, and drugs such as nicotine and dopamine antagonists. In our case, since periodic circulatory fluctuations started after tracheal intubation, it is possible that the blood flow of the tumor changed due to changes in hemodynamics caused by the anesthesia-inducing drugs and stimulation of the tracheal intubation [10]. On the other hand, stabilizing circulating blood volume has been reported as a possible solution to interrupt the hypertensive cycle [2]. A decrease in intravascular volume due to preoperative hemodialysis and induction of anesthesia may have triggered periodic abnormal hypertension.

In our patient, nicardipine and landiolol were administered for blood pressure fluctuations; however, the response was poor. Hemodynamic control was considered impossible due to the release of very strong catecholamines during the periodic fluctuations. Alpha blockers have been reported to stabilize hypertension due to pheochromocytoma. Our patient developed hemodynamic fluctuations after two inductions of anesthesia, but the degree of fluctuation was smaller during the second surgery than during the first. It is possible that the degree of periodic fluctuation was reduced via the oral administration of doxazosin after the first surgery. In general, administration of an alpha blocker for 1–2 weeks is recommended before pheochromocytoma resection for intraoperative hemodynamic stabilization. In this case, the alpha blocker was also effective against periodic fluctuations.

In conclusion, periodic abnormal circulatory fluctuations were observed during anesthesia due to periodic catecholamine release from the pheochromocytoma. The cause of rare periodic blood pressure fluctuations in patients with pheochromocytoma is thought to be related not only to the cycle of sympathetic and parasympathetic reflexes but also to the periodic secretion of catecholamines.

## Data Availability

The data used in this case report are available from the corresponding author upon reasonable request.

## Abbreviations

HR: Heart rate
SBP: Systolic blood pressure
DAP: Diastolic blood pressure
NIBP: Noninvasive blood pressure
Hb: Hemoglobin
BUN: Blood urea nitrogen
Cre: Creatinine
